# Identification of Theaflavin-3,3’-Digallate as a Novel Zika Virus Protease Inhibitor

**DOI:** 10.3389/fphar.2020.514313

**Published:** 2020-10-21

**Authors:** Xiangling Cui, Rui Zhou, Chenchao Huang, Rongyu Zhang, Jing Wang, Yongxin Zhang, Jiwei Ding, Xiaoyu Li, Jinming Zhou, Shan Cen

**Affiliations:** ^1^Institute of Medicinal Biotechnology, Chinese Academy of Medical Science, Beijing, China; ^2^Key Laboratory of the Ministry of Education for Advanced Catalysis Materials, Department of Chemistry, Zhejiang Normal University, Jinhua, China; ^3^Drug Discovery & Innovation Center, College of Chemistry and Life Sciences, Zhejiang Normal University, Jinhua, China; ^4^CAMS Key Laboratory of Antiviral Drug Research, Institute of Medicinal Biotechnology, Peking Union Medical College, Chinese Academy of Medical Sciences, Beijing, China; ^5^Beijing Institute of Tropical Medicine, Beijing Friendship Hospital, Capital Medical University, Beijing, China

**Keywords:** Zika virus, natural active compound, screen, anti-virus, protease

## Abstract

Mounting evidence indicates that Zika virus (ZIKV) is closely related to neurological disorders such as microcephaly and Guillain-Barré syndrome. There are currently no effective vaccines and FDA-approved inhibitors against ZIKV infection. The flaviviral heterodimeric serine protease NS2B-NS3 plays an essential role in ZIKV maturation and replication, thus becoming a promising target in anti-ZIKV therapy. Herein, we developed a fluorescence-based screening assay to search for inhibitors targeting the ZIKV NS2B-NS3 protease (ZIKVpro), and identified theaflavin-3,3’-digallate (ZP10), a natural active compound derived from black tea, as a potent ZIKV protease inhibitor *in vitro* (IC_50 =_ 2.3 μM). ZP10 exhibited dose-dependent inhibitory effect on ZIKV replication (EC_50 =_ 7.65 μM). Western blot analysis suggested that ZP10 inhibited the cleavage processing of viral polyprotein precursor in cells either infected with ZIKV or expressing minimal self-cleaving proteinase NS2B-3 protease, resulting in inhibition of virus growth. Moreover, ZP10 was showed to directly bind to ZIKVpro, and a docking model further revealed that ZP10 interacted with several critical residues at the proteolytic cavity of the ZIKVpro. This study highlights that ZP10 has anti-ZIKV potency through ZIKVpro inhibition, which indicates its potential application in anti-ZIKV therapy.

## Introduction

The recent outbreak of Zika virus (ZIKV) belonging to the family flaviviridae has posed a threat to human health. In the early stage, ZIKV was neglected because of its small geographical distribution and mild clinical disease (Gatherer and Kohl, 2016; Salvador and Fujita, 2016; Holbrook, 2017). However, although most infections are asymptomatic, its rapid spread in multiple regions, sexual and vertical human-to-human transmissions and its association with severe neurological disorders (Guillain-Barré syndrome and microcephaly) prompted World Health Organization (WHO) to declare ZIKV as a Public Health Emergency of International Concern (Heymann et al., 2016). Nevertheless, no vaccine or therapeutic agent has been clinically approved for preventing or controlling ZIKV infection so far. This unmet medical need has motivated academia and industry to develop countermeasures. To date, several promising ZIKV vaccine candidates have already entered clinical trials. In contrast, antiviral development of ZIKV is still only in its infancy. Three strategies have been developed for ZIKV drug discovery, including (1) repurposing of clinically approved drugs, (2) viral replication-based phenotypic screening for inhibitors, and (3) targeted drug discovery of viral proteins (Zou and Shi, 2019). Considering the urgent need for anti-ZIKV drugs, repurposing of approved drugs appears to be a viable and immediate solution. For instance, anti-malaria drug chloroquine (CQ) (Shiryaev et al., 2017), sofosbuvir (Bullard-Feibelman et al., 2017; Sacramento et al., 2017), and ribavirin (Kamiyama et al., 2017) showed great potential to be repurposed as antiviral drug for ZIKV treatment and prophylaxis.

**Figure 1:**
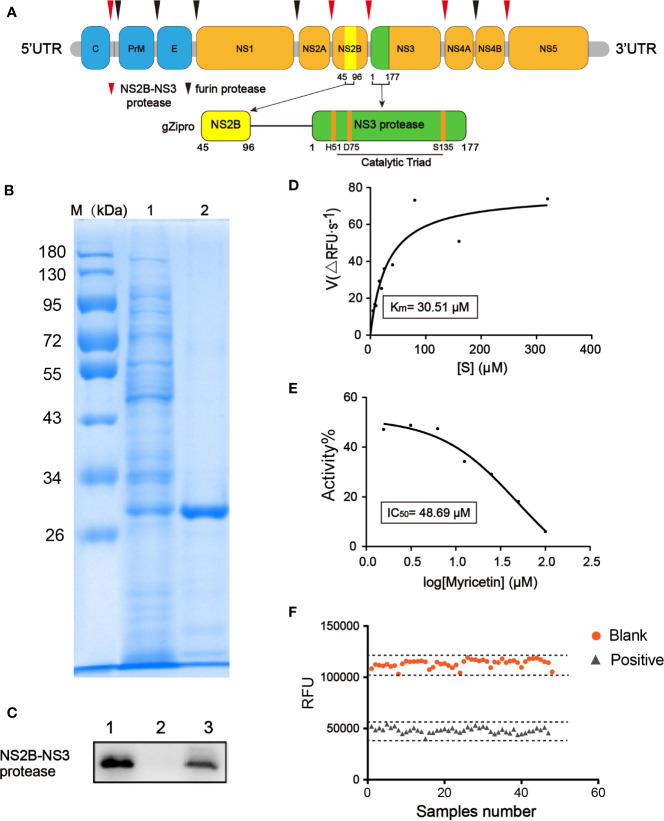
Development of fluorescence-based screening assay for ZIKVpro inhibitors. **(A)** From top to bottom, Zika virus (ZIKV) polypeptide, a glycine rich G_4_SG_4_ artificial linker (gZiPro). **(B)** SDS-PAGE of the purified ZIKVpro expressed in *E.coli* BL21(DE3). The *lane 1* refers to the cell lysate with 0.5 mM IPTG induction to overexpress of ZIKVpro; *lane 2* refers to purified ZIKVpro after Ni–NTA column chromatography. **(C)** Western blot analysis of purified ZIKVpro using anti-His-tag antibodies. The *lane 1* refers to the cell lysate with 0.5 mM IPTG induction to overexpress of ZIKVpro; *lane 2* refers to sample flowing through column without binding; *lane 3* refers to purified ZIKVpro after Ni–NTA column chromatography. **(D)** Michaelis-Menten curve of ZIKVpro with substrate from 5 to 320 µM. According to the analysis, the K_m_ value was 30.51 μM. **(E)** The inhibition of ZIKVpro by myricetin. Two-fold dilutions (from 100 μM to 1.5625 μM) were used. The protease activity of the DMSO control was set as 100%. **(F)** Determination of Z’ factor of the fluorescence-based screening assay. One-half plate of the active ZIKVpro was incubated in 100 μM of positive compound Myricetin or 1% DMSO for 1 h at 37 °C. The reaction was started by addition of Bz-nKKR-AMC. After 1 h, fluorescence intensity was measured and Z’ factor was calculated as described.

ZIKV encodes a single polyprotein that can be cleaved into structural (capsid, pre-membrane and envelope) and nonstructural proteins (NS1, NS2A, NS2B, NS3, NS4A, NS4B, and NS5) essential for viral replication, virion assembly, and evasion from the host defense mechanisms. This essential cleavage is executed by host and viral proteases at the membrane of the endoplasmic reticulum (Kang et al., 2017). ZIKV NS2B-NS3 protease (ZIKVpro) is responsible for all cytoplasmic cleavages including at junctions between capsid/membrane, NS2A/NS2B, NS2B/NS3, NS3/NS4A, and NS4B/NS5 proteins (**Figure 1A**) (Luo et al., 2015; Nitsche, 2019). The NS3 N-terminal domain is a member of the trypsin/chymotrypsin protease superfamily with an absolutely conserved catalytic triad H51, D75, and S135 (Li et al., 2017; Mahawaththa et al., 2017). The small transmembrane protein NS2B anchors NS3 to the endoplasmic reticulum membrane, and together they form an active enzyme for substrate recognition and catalysis (Phoo et al., 2016; Zhang et al., 2016; Nitsche, 2018). Given that antiviral drugs targeting the NS3/NS4A protease have successfully been used to treat hepatitis C virus (HCV) infections, ZIKVpro is regarded as a particularly promising therapeutic target for antiviral development (Lee et al., 2013; Lee et al., 2017). Generally, the substrate peptide is a suitable starting point for the drug discovery of ZIKVpro (Gruba et al., 2016; Li et al., 2018). Nevertheless, this strategy remains the key drawbacks of peptidic inhibitors, such as low cellular activity and bad oral pharmacokinetics (Kang et al., 2017). Consequently, developing novel classes of non-peptide and peptidomimetic inhibitors against ZIKVpro has been of great interest for researchers from both academia and industry.

Natural-derived products has received growing attention for their huge potential to boost the development of new medicines (Cragg and Newman, 2013; Calixto, 2019). To date, few plant-derived natural products have been identified as inhibitors against ZIKVpro. The first reported natural product is myricetin, a polyphenol compound of flavones, showed inhibiting activity against ZIKVpro with the IC_50_ of 22.0 μM (Lim et al., 2017). Amrita Roy et al. then identified five flavonoids and one natural phenol rich in edible plants as ZIKVpro inhibitors with IC_50_ ranging from 1.3 μM for Myricetin to 56.3 μM for Apigenin (Roy et al., 2017). However, the anti-ZIKV activities of the above compounds have not been reported. In this work, we utilized a fluorescence-based high-throughput screening assay to search for inhibitors targeting the ZIKVpro. Theaflavin-3,3’-digallate (ZP10) was found to potently inhibit the ZIKVpro *in vitro*, also inhibit ZIKV replication in a dose-dependent manner. Further overexpressing the minimal proteinase NS2B/3 protease in HEK293T cells suggested that ZP10 can inhibit the activity of NS2B-3 protease, which was correspond with the increase of ratio of ZIKV polyprotein precursor and NS2B-3 precursor. Moreover, ZP10 was identified directly binding to ZIKVpro, and the binding mode was further predicted through molecular modeling. The findings of this study demonstrated for the first time that ZP10 has potential to target ZIKVpro to produce a protective effect *in vitro* against the infection of ZIKV, which suggests its potential application in anti-ZIKV therapy.

## Results

### Development of Fluorescence-Based Screening Assay for ZIKVpro Inhibitors

We constructed a ZIKVpro expressing vector containing NS2B (residues 45–96) and NS3pro (residues 1-177) linked by a (Gly)_4_-Ser-(Gly)_4_ sequence, followed by a poly-histidine tag in C-terminal ends, which was extensively used for functional and structural characterization of flaviviral NS2B-NS3pro complexes (Kang et al., 2017; Nitsche, 2019) (**Figure 1A**). The ZIKVpro enzyme was expressed in *E.coli* BL21(DE3) and then purified by a His-trap excel column (GE healthcare). SDS-PAGE analyses revealed a protein band of approximately 30 kDa with over 80% in purity (**Figures 1B, C**).

Similar to proteases from other flavivirus such as dengue virus (DENV) and West Nile virus (WNV), ZIKVpro recognizes and cleaves Lys-Arg, Arg-Arg, Arg-Lys or Gln-Arg motifs (Gruba et al., 2016). Therefore, we used benzoyl-norleucine-lysine-lysine-arginine 7-amino-4-methylcoumarine (Bz-nKKR-AMC), one of the commercially available substrates for flaviviruses protease, as the substrate to assess the kinetic parameter of ZIKVpro (Phoo et al., 2016), resulting in a K_m_ value of 30.51 µM (**Figure 1D**). Based on the determined K_m_ value, we decided to use 50 µM substrate for high-throughput screening assay. Myricetin was reported to inhibit the activity of ZIKVpro in a dose-dependent manner (IC_50 =_ 48.69 µM) (Roy et al., 2017), which was used as the positive control. As shown in **Figure 1E**, myricetin exhibited a robust dose-response to inhibit ZIKVpro in the screening assay.

Next, we determined the key performance parameters of fluorescence-based screening assay for ZIKVpro inhibitors in a 96-well plate. One-half plate of the active ZIKVpro was incubated in 100 μM of positive compound Myricetin or 1% DMSO for 1 h at 37 °C. The reaction was triggered by addition of Bz-nKKR-AMC. The Z’ factor of the assay is 0.7 (**Figure 1F**), and signal to noise ratio(S/N), CV% are 14.23 and 3.26%, respectively, suggesting a high reproductivity and feasibility of the assay under the selected experimental condition.

### Preliminary Screening and Confirmation of Hits

**Figure 2:**
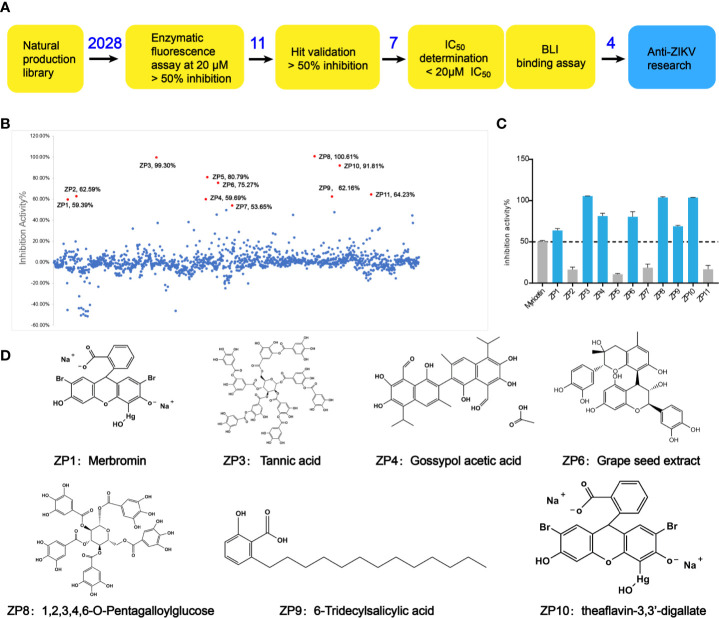
Preliminary screening and hit validation. **(A)** Schematic of high-throughput screening and hit validation process. **(B, C)** Primary high-throughput screening and hit validation. Compounds were added at a concentration of 20 μM and protease activity relative to the DMSO control was measured. No inhibitor (No Inh) was set as 100%. **(D)** Schematic formulas of seven hits showing more than 50% inhibition activity against ZIKVpro.

**Figure 3:**
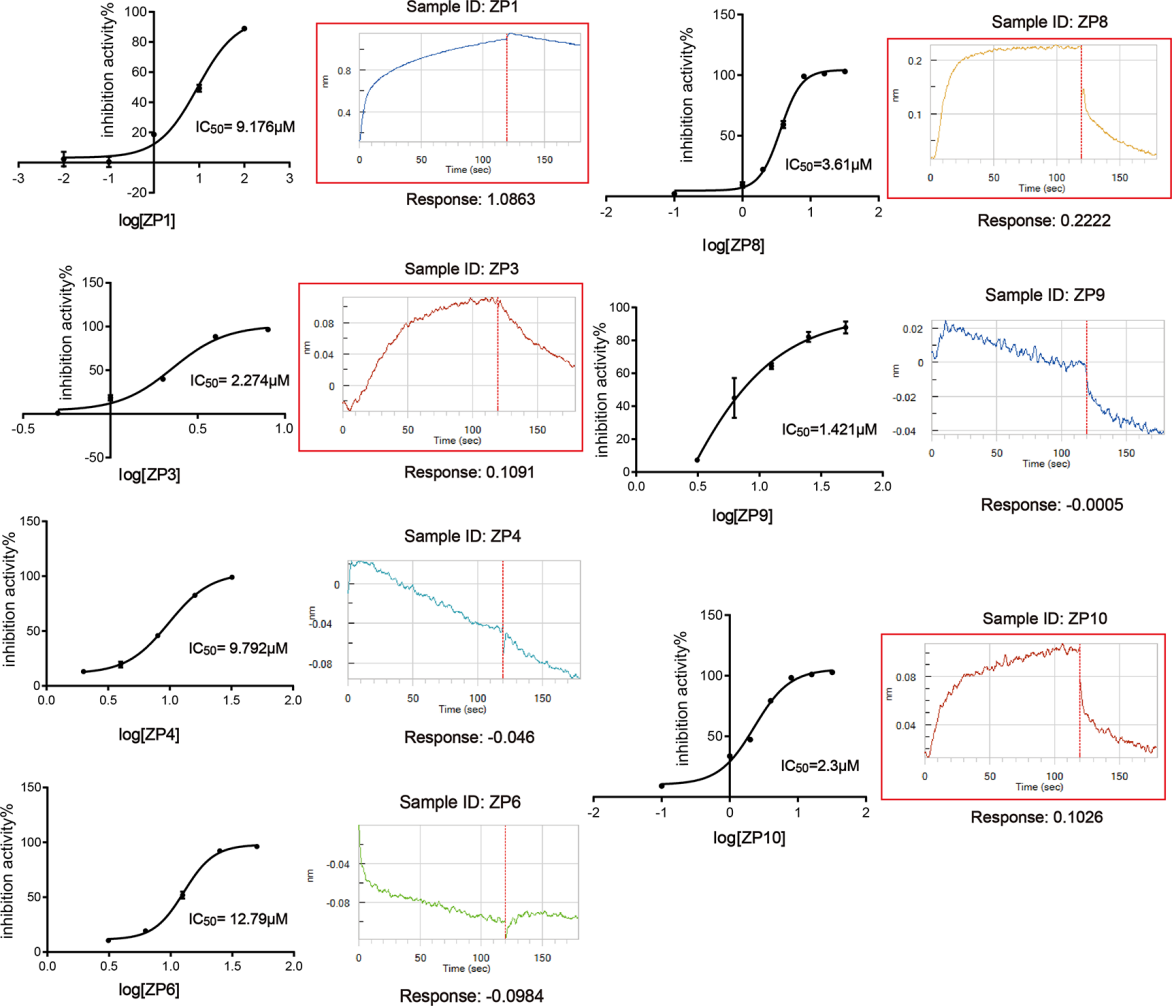
Hit Confirmation. Concentration curves of *in vitro* protease activity inhibited by selected compounds. The results of Octet binding assay using biotinylated ZIKVpro indicated four of the compounds binding to ZIKVpro at 50 µM, including merbromin (ZP1), tannic acid (ZP3), 1,2,3,4,6-O-Pentagalloylglucose (ZP8), and theaflavin-3,3’-digallate (ZP10).

Through the screening model, the screening of a natural compound library (TargetMol) was performed to get ZIKVpro inhibitors. The overall workflow was shown in **Figure 2A**. The preliminary screen yielded 11 hits (**Figure 2B**). The confirmed screening got seven of the initial hits, which exhibited more than 50% inhibition at a concentration of 20 µM (**Figures 2C, D**). Then we observed that all the seven compounds inhibited the ZIKVpro activity in a dose-dependent manner with IC_50_ ranging from 1.4 μM for ZP9 to 9.8 μM for ZP4 (**Figure 3**). Furthermore, Octet binding assay using biotinylated ZIKVpro showed four of the compounds with a direct binding to ZIKVpro at 50 μM (**Figure 3**), including merbromin (ZP1), tannic acid (ZP3), 1,2,3,4,6-O-pentagalloylglucose (ZP8) and theaflavin-3,3’-digallate (ZP10). The abilities to inhibit ZIKVpro enzymic activity and bind to ZIKVpro suggest that these four hits possess the potential for further development as ZIKVpro inhibitors.

### ZP10 Is a Potent Inhibitor Against ZIKV Replication

**Figure 4:**
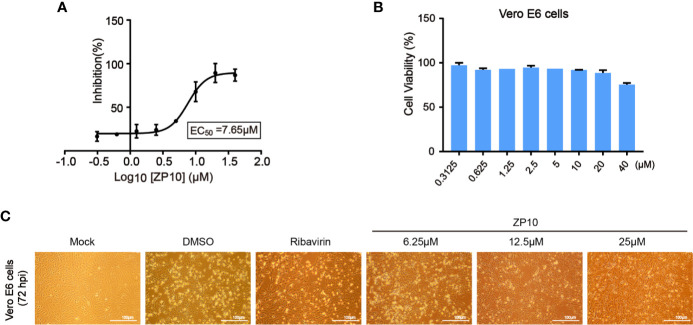
ZP10 is a potent inhibitor against Zika virus (ZIKV) infection with low cytotoxicity. **(A)** Dose-dependent inhibition of ZIKV by ZP10 in Vero E6 cells. **(B)** Vero E6 cells were treated with ZP10 at indicated concentrations (0.3125~40 μM). The cell viability was measured by CCK-8 assay. **(C)** Cell morphology was assessed using an AMG EVOS micro-scope with an attached camera by comparing treated and untreated samples.

To assess if these compounds were effective against the replication of ZIKV, a cell-based assay was utilized using African monkey kidney cell line (Vero E6) infected with ZIKV, which measures the protection of cell viability against ZIKV-induced cytopathic effect (CPE) as a read-out. The result showed an EC_50_ value of 7.65 μM for ZP10 (**Figure 4A**), suggesting a potent anti-ZIKV activity, while other three compounds had no appreciable effect, e.g., less than 30% CPE protection at concentration of 10 μM (data not shown). In agreement with above result, we found that ZIKV-induced CPE was attenuated to a large extent at 72 hpi in Vero E6 cells treated with ZP10 in a dose dependent manner (from 6.25 µM to 25.0 µM) (**Figure 4C**). Ribavirin, a previously identified inhibitor with potent suppression of the replication of ZIKV (Kamiyama et al., 2017; Kim et al., 2018), showed a similar effect. Furthermore, we measured the viability of Vero E6 cells in the presence of a range of ZP10 using commercially available CCK-8, and found minor cytotoxicity up to 40 μM (**Figure 4B**), suggest no cytotoxicity effect involved in anti-ZIKV activity of ZP10. These results together suggest that ZP10 is a potent inhibitor against ZIKV replication.

**Figure 5:**
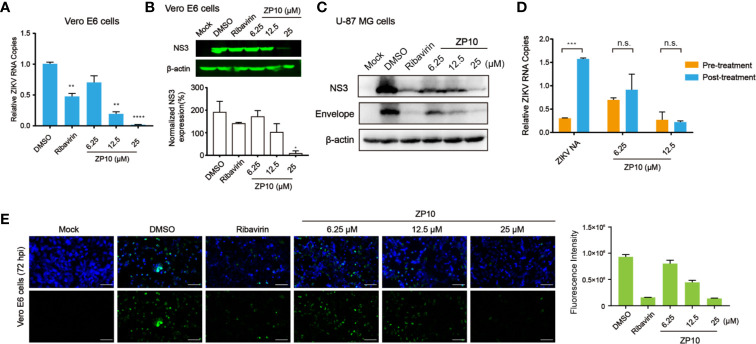
ZP10 can inhibit the post-entry events of the Zika virus (ZIKV) replication cycle from gene transcription and translation levels. **(A)** qRT-PCR analyses of inhibition of viral RNA from ZP10-treated and ZIKV-infected Vero E6 cells. **p < 0.01, ****p < 0.0001, by one-way ANOVA. Data were shown as the mean ± SD (N = 3). **(B)** Western Blot analysis of inhibition of ZIKV NS3 expression from ZP10-treated and ZIKV-infected samples in Vero E6 cells. NS3 expression was normalized to the β-actin loading control. *p < 0.05. Data were shown as the mean ± SD (N=3). **(C)** Western Blot analysis of inhibition of viral protein (NS3 and envelope) from ZP10-treated and ZIKV-infected U-87 MG cells. **(D)** qRT-PCR analysis of inhibition of viral RNA from ZP10-treated and ZIKV-infected Vero E6 cells before and after the stage of viral adsorption. ZIKV Neutralizing antibody (ZIKV NA) was treated as a positive control which can inhibit the viral adsorption events (0–1 hpi). ***p < 0.001. Data were shown as the mean ± SD (N=3). **(E)** Left panel: Representative images of immunostaining of infected and uninfected Vero E6 cells with or without ZP10 treatment. Alexa‐Fluor 488 (green) was used as the secondary antibody to detect ZIKV envelope proteins. Right panel: the quantification of green fluorescence intensity. n.s., no significant difference was detected.

To further inspect the anti-ZIKV activity of ZP10, we performed quantitive RT-PCR (qRT-PCR) and western blot analysis to assess its effect on RNA and protein expression of ZIKV. We used Vero E6 cells as host cells which is of high susceptibility to ZIKV. As expected, ZP10 markedly reduced viral RNA copy numbers (**Figure 5A**) and NS3 protein expression in a dose-dependent manner (**Figure 5B**). Moreover, immunofluorescence assay (IFA) for ZIKV envelope proteins indicated that ZP10 markedly reduced the expression of ZIKV envelope proteins at concentration of 12.5 µM and 25.0 µM, which is comparable to that of ribavirin (**Figure 5E**). Additionally, ZIKV also preferentially infected and killed glioblastoma stem cells relative to normal neuronal cells (Zhu et al., 2017; Zhu et al., 2020). Therefore, we further evaluated the drug efficacy in human glioblastoma cell lines U-87 MG (Tricarico et al., 2019). The results indicated that ZP10 also considerably reduces viral protein expression in U87 MG in a dose-dependent manner (**Figure 5C**). Collectively, these experiments confirmed ZP10’s efficacy for controlling ZIKV infection in both cells.

Next, we performed a time-of-addition assay as described previously (Wang et al., 2016) to explore any possible effect of ZP10 on viral entry. The result showed a similar inhibitory effect on ZIKV replication with ZP10 treatment either before or after the virus adsorption stage (0–1 hpi), indicating no effect of ZP10 on ZIKV entry (**Figure 5D**). On the contrary, the treatment with neutralizing antibodies targeting ZIKV after viral entry stage significantly lost its ability to inhibit ZIKV replication (**Figure 5D**). Taken together, these results indicated that ZP10 is a potent inhibitor targeting the post-entry step of ZIKV replication cycle.

### Inhibition of Viral Polyprotein Precursor Processing

**Figure 6:**
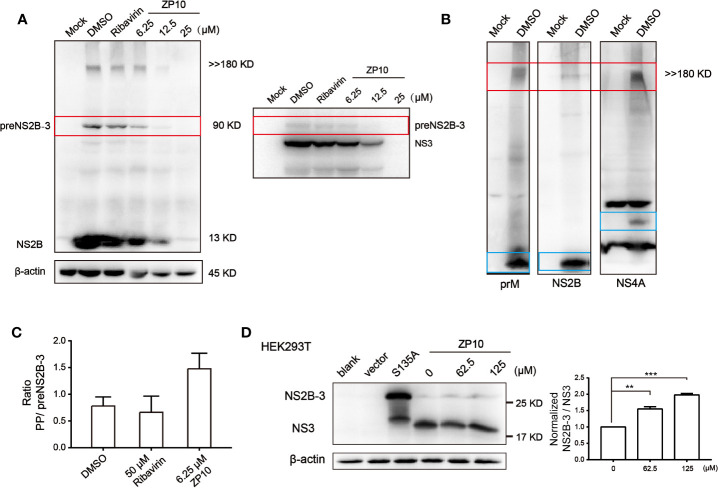
ZP10 has potential to disrupt the cleavage of viral polyprotein precursor. **(A)** Left panel: Western Blot analysis of inhibition of Zika virus (ZIKV) NS2B expression from ZP10-treated and ZIKV-infected samples in Vero E6 cells. Right panel: Western blot to confirm the intermediate of ZIKVpro from the course of polyprotein cleavage. **(B)** Western blot to confirm polyprotein. Four antibodies could detect a band of high molecular weight protein (>> 180 kDa) including anti-prM, NS2B and NS4A antibodies. **(C)** The increase of ratio of ZIKV polyprotein precursor and preNS2B-3 indicated the PP accumulation in the presence of ZP10. The targeted protein expression was normalized to the β-actin loading control. Data were shown as the mean ± SD (N=3). **(D)** Left panel: Western blot analysis of inhibition of ZIKVpro activity on HEK293T cells by ZP10. Minimal proteinase was overexpressed in the presence of increasing concentrations of ZP10. Right panel: The increase of ratio of NS2B-3/NS3 (normalized by DMSO control) indicated the accumulation of NS2B-3 precursor in the presence of ZP10. The targeted protein expression was normalized to the β-actin loading control. **p < 0.01, ***p < 0.001. Data were shown as the mean ± SD (N=3).

Previous enzyme kinetics inhibition analysis indicated that ZP10 potently inhibits the catalytic activity of ZIKVpro. Herein, we further checked whether ZP10 inhibits the cleavage processing of polyprotein precursor (PP) catalyzed by ZIKVpro during ZIKV infection. Western blot analysis of ZIKV-infected cell lysate probed with anti-NS2B antibody showed a distinct band of an intermediate (~90 kDa) and a high molecular weight (MW) protein (>> 180 kDa) (**Figure 6A** left panel). The intermediate was reacted with both anti-NS2B and anti-NS3 antibody (**Figure 6A**), and most likely represents the unprocessed NS2B-NS3 complex intermediate, termed as preNS2B-3, with a theoretical MW of approximate 90 kDa. The high MW product likely represents the unprocessed viral PP (theoretical MW: 379 kDa) which was recognized by other antibodies specific for prM and NS4A of ZIKV, respectively (**Figure 6B**). Of note, Matthew Brecher et al. also detected such a high MW band by anti-NS3 antibody (Brecher et al., 2017), and then further characterized this protein through mass spectrometry analysis. There were peptides corresponding to the ZIKV envelope, NS3 and NS5 proteins being identified. Therefore, it is reasonable to deduce that this high MW band was the unprocessed viral PP. Upon ZP10 treatment, especially at 6.25 µM, the amount of PP kept unchanged, while the intermediate and the fully-cleaved products such as NS2B were significantly reduced (**Figure 6A**), suggesting an impaired cleavage processing of PP. In support of the conclusion, we found an increase in the ratio of PP/preNS2B-3 in the ZP10-treated group compared to ribavirin-treated group (**Figure 6C** and **Supplementary Figure 1A**).

To further investigate whether ZP10 inhibits ZIKVpro in cells, we used a minimal self-cleaving proteinase NS2B-3pro which is extensively used for functional and structural study of ZIKVpro (Phoo et al., 2016). This construct consists of the required cofactor domain of NS2B (amino acids 45 to 96), the last five residues of NS2B including enzymatic cleavage site (amino acids 126 to 130), and the protease domain of NS3 (amino acids 1 to 177). Meanwhile, a proteinase active-site mutation, NS3-S135A, caused this protein to be produced as a precursor only and also provides a marker for intact NS2B-3pro. Wint Wint Phoo et al. determined the exact MW of NS2B/3pro (25.71 kDa) and free NS3 protease (19.03 kDa) using matrix assisted laser desorption ionization, which are accord with the MW of protein we detected by NS3 antibodies (Phoo et al., 2016). We noticed that NS2B-3pro was efficiently expressed and cleaved into free NS3 after 48 h in HEK293T cells. ZP10 inhibited this cleavage, as seen by the accumulation of intact NS2B-3pro with increasing concentration (**Figure 6D** and **Supplementary Figure 1B**). Collectively, these data indicated that ZP10 inhibited the cleavage processing of PP and its antiviral activity likely result from impairing the ZIKVpro catalytic activity.

### ZP10 Directly Binds to ZIKV NS2B-NS3 Protease

**Figure 7:**
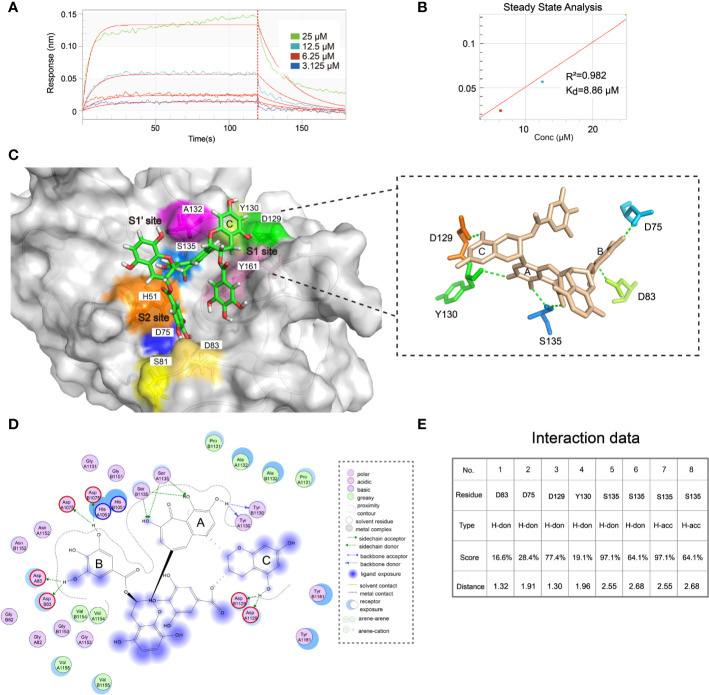
ZP10 directly binds to Zika virus (ZIKV) NS2B-NS3 protease. **(A, B)** The binding kinetics of immobilized biotinylated gZiPro against ZP10 was measured using bio-layer interferometry optical analytical technique. The Langmuir’s 1:1 binding model was applied to fit the interferometry data. **(C)** Predicted poses of compound ZP10 in the binding pocket of the gZiPro structure (PDB code: 5LC0). ZP10 is in stick representation with carbons colored green. Right panel: The 3D interaction diagram of ZP10 with ZIKVpro. **(D, E)** The 2D interaction diagram of ZP10 with ZIKVpro.

To further evaluate the binding affinity of ZP10, we performed bio-layer interferometry binding assays (BLI) using biotinylated ZIKVpro on super streptavidin sensors (SSA) for ZP10. As shown in **Figures 7A, B**, ZP10 bond to ZIKVpro in a dose-dependent manner. According to the steady state analysis, the binding affinity K_d_ of ZP10 was 8.86 µM. These data further confirmed that ZP10 was capable of binding to ZIKVpro directly.

Then, we further investigated the binding mode of ZP10 with ZIKVpro through molecular docking module in MOE. The best fit docking pose was shown in **Figure 7C**. ZP10 was predicted to interact with a number of residues of ZIKVpro including D83, D75, D129, Y 130, and S135 *via* hydrogen bonds (**Figures 7C–E**). The ring C of ZP10 was sandwiched by residues A132 and Y161 in the S1 pocket of the ZIKVpro catalytic cavity *via* hydrogen bond interaction with D129 (**Figures 7C, D**). The ring A formed hydrogen bond with Y130 and S135 respectively and occupied pocket S1’ of the ZIKVpro catalytic cavity which was formed by residues V36, H51, K54, and S135. The ring B interacted with the main chain carbonyl oxygen of D75 and D83 and bond to the substrate binding pocket (S2 site) of the ZIKVpro catalytic cavity which was formed by S81, D83 of NS2B and H51, D75 of NS3. Taken together, the results of the binding assay and the molecular docking further supported that ZP10 was a potent inhibitor against ZIKVpro.

## Discussion

The research progress of inhibitors against ZIKVpro has been described and comprehensively reviewed (Kang et al., 2017). These compounds can be classified into two groups: substrate-derived peptides/peptidomimetics and small molecules without substrate character. Growing evidence demonstrates that the former group shows an exquisite high affinity for ZIKVpro but limited drug-likeness (Nitsche, 2019). Small molecular inhibitors may address such concerns. However, to date, only a few plant-derived natural products have been identified as ZIKVpro inhibitors. Among others myricetin is the first reported, and also the best plant-derived ZIKVpro inhibitor. However, the anti-ZIKV activities of myricetin have not been reported yet. Our screening assay identified one potential inhibitory compound, ZP10, standing out as a novel small molecular inhibitor against ZIKVpro (IC_50 =_ 2.3 μM) with potent anti-ZIKV activity (EC_50 =_ 7.65 μM) and low cytotoxicity. ZP10 was reported to have antiviral activity against HSV and HIV-1 (Yang et al., 2012; de Oliveira et al., 2015; Mostashari-Rad et al., 2019). As for HSV-1, ZP10 inhibited its entry into target cells by interfering with the binding/adsorption of virions to the cellular receptor (de Oliveira et al., 2015). About HIV-1 infection, ZP10 played as a potent inhibitor of gp41 which is very critical for the progression of HIV-1 virion and host cell fusion (Mostashari-Rad et al., 2019). In this study, we showed that ZP10 inhibited ZIKVpro and impaired a post-entry step of ZIKV replication, suggesting its new antiviral mechanism differed from previous work. Furthermore, it can significantly reduce viral RNA copy numbers and NS3 protein expression in a dose-dependent manner. Results from western blot and qRT-PCR are consistent with our hypothesis that ZP10 suppresses viral protease function, leading to reduced expression of viral NS2B and NS3 protein. Particularly it inhibited ZIKV growth not only in Vero E6 cells but also in human glioblastoma cell lines U-87 MG.

Furthermore, the ratio of ZIKV polyprotein precursor and preNS2B-3 suggested that ZP10 inhibits the cleavage processing of viral polyprotein precursor, eventually leading to inhibition of virus growth. However, infectious virus-based assay has to face problems introduced by viral replication cycle, which make it less accessible to detect the change of viral polyprotein precursor promptly. In this scenario, we used a minimal self-cleaving proteinase NS2B-3pro to determine the effect of ZP10 on ZIKVpro directly. However, ZP10 exerted its inhibition on ZIKVpro self-cleavage in a high concentration (125 μM), compared with viral inhibition (EC_50 =_ 7.65 μM) and cleavage of small substrates *in vitro* (IC_50_ = 2.3 μM). Notably, dengue protease inhibitor ARDP006 was about 100-fold more effective at inhibiting viral growth (EC_50_ = 4.2 μM) than the enzymatic activity of the viral proteinase (IC_50_ = 432 μM) (Constant et al., 2018). Moreover, another ZIKVpro-targeted molecule NSC135618 (Brecher et al., 2017) was also determined its viral and intramolecular cleavage IC_50_ value, which is 1.7 μM and 490 μM, respectively (Constant et al., 2018). The discrepancy in the effective concentrations found for ZP10 might result from either different sensitivity of ZIKV protease in the virus content and in protease assay, or an amplification effect of inhibited protease activity on viral replication. Additionally, it is more likely that ZP10 indeed inhibits ZIKV growth through inhibition of the ZIKVpro, but by potentially complex mechanisms.

Additionally, we performed bio-layer interferometry binding assays (BLI) to determine the binding affinity K_d_ of ZP10 to ZIKVpro (K_d_ = 8.86 μM), indicating that ZP10 was capable of binding to ZIKVpro directly. Finally, we further investigated the binding mode of ZP10 with ZIKVpro through molecular docking module. The best fit docking pose showed that ZP10 can interact with a number of residues in the ligand pocket of ZIKVpro, including D83, D75, D129, Y130, and S135 *via* hydrogen bonds. It is worth noting that D75 and S135 of ZIKVpro involving in ZP10 binding are vital to enzyme activity. The cofactor NS2B and NS3 together form an active enzyme with a substrate binding pocket which has five subsites (S1, S1’, S2, S3, S4) (Zhang et al., 2016). The result of molecular docking simulation showed that ZP10 was predicted to occupy S1, S2, and S1’ sites. Previous reports identified that S2 pocket of ZIKVpro is critical for substrate recognition and catalytic activity (Li et al., 2017). However, the docking study was only the prediction of the potential binding pose of ZP10 in the catalytic site. To substantiate the docking result, structural or competition studies are necessary to further confirm the binding mode of ZP10 to ZIKVpro. Collectively, it is reasonable to assume that ZP10 is likely to target the protease of ZIKV and the inhibitory potency against ZIKV infection was mainly through the disruption of ZIKV polyprotein cleavage (Lei et al., 2016; Rut et al., 2017).

However, ZP10 is one of the four main theaflavins in black tea, which is produced by the oxidative dimerization of epicatechin gallate (ECG) and (−)-epigallocatechin-3-gallate (EGCG), which are the major catechins found in green tea (Gao et al., 2016). Consequently, ZP10 possesses many phenol hydroxyl, which may lead to non-specific interaction with drug target. Despite the above findings, we still need alternate strategies to explore the specificity of ZP10 to ZIKVpro and determine the interaction between ZP10 and ZIKVpro. Overall, ZP10 is supposed to be a good starting scaffold for novel ZIKVpro inhibitors development, and structural optimization of ZP10 is expected to result in more reliable ZP10 derivatives against ZIKV based on the predicted binding mode.

## Materials and Methods

### Compounds

All compounds were obtained from the TargetMol natural compound library, typically in >95% purity. For *in vitro* and cellular assays, the solid compounds were reconstituted with DMSO to stock concentrations of 10 mM. All compounds were stored at -20 °C until use.

### Plasmid Construction and Purification of Zika NS2B-NS3 Protease

The cDNA coding for residues 45–96 of ZIKV NS2B and the N terminus of NS3 (residues 1–177) with a GGGGSGGGG artificial linker was inserted into the *NcoI* and *XhoI* sites of pET28a vector. pET28a-NS2B-NS3 protease was transformed into *E.coli* BL21 (DE3) which was then grown in LB supplemented with kanamycin (50 μg/ml) at 37 °C when OD_600_ reached 0.8. The cells were then induced with 0.5 mM IPTG for 16 h at 16 °C. Harvested cells were lysed by homogenization in equilibration buffer (50 mM Tris and 500 mM sodium chloride). The His-tag fused ZIKVpro was purified by a Histrap excel column (GE healthcare) with a stepwise gradient of buffer B (50 mM Tris (pH7.5), 500 mM NaCl, 500 mM imidazole, 5% glycerol, and 5 mM β-mercaptoethanol). The pooled ZIKVpro was then dialyzed with buffer C (50 mM Tris, 150 mM NaCl, pH 7.5). The concentration of protein was determined using the BCA protein assay. Purified protein was confirmed by 10% SDS-PAGE.

### Kinetic Parameter Determination

Proteolytic activity of NS2B-NS3 protease was measured using benzoyl-norleucine-lysine-lysine-arginine 7-amino-4-methylcoumarine (Bz-nKKR-AMC). Bz-nKKR-AMC substrate with starting concentration of 300 μM was serially diluted two times in reaction buffer (50 mM Tris-HCl, pH 7.5, 150 mM NaCl) and added to 96-well black plate (Costar, USA) with 150 nM protein diluted in the same buffer. For kinetics measurements, fluorescence readings were measured at 30 s interval for 5 min using Enspire (Perkin Elmer) at excitation wavelength (λ_ex_) at 360 nm and emission wavelength (λ_em_) at 460 nm. Assays were carried out as triplicates at 37 °C. Michaelis-Menten constant (K_m_) was calculated by graphpad prism5.0.

### Primary Assay for Compound Screening

For initial screening, each compound was dissolved in dimethyl sulfoxide (DMSO) to obtain 20 mM stock solution. The NS2B/NS3 protease at concentration of 150 nM were incubated in 20 µM compounds in buffer containing 50 mM Tris-HCl, 150 mM NaCl, pH 7.5 for 1 h at 37 °C in 96-well plate. The reaction was started by addition of Bz-nKKR-AMC substrate at 50 µM and fluorescence readings were measured at 30 s intervals over 10 min using Enspire (Perkin Elmer, USA) at excitation wavelength(λ_ex_) at 360 nm and emission wavelength(λ_em_) at 460 nm. Assays were carried out as triplicates at 37 °C.

Compounds were incubated with the linked ZIKVpro (150 nM) for 1 h, prior to addition of the AMC substrate (50 μM). The protease activity of the DMSO control was set as 100%. The protease activities with compounds were set as percentage of the DMSO control. Data was fitted using the log(inhibitor) vs. response - variable slope (four parameters) model within the graphpad prism 7.0 software.

$$\text{Inhibition}\;\text{activity}=100-\left[\frac{S–S_{0}}{C-C_{0}}\right]\times 100\%$$

S: the RFUs of the test sample (enzyme, inhibitor, buffer, and substrate) after 10 min of reaction

S_0_: the RFUs of the tested samples at time zero.

C: the RFUs of the control (enzyme, buffer, and substrate) after 10 min of reaction

C_0_: the RFUs of the control at time zero

### Bio-Layer Interferometry (BLI)

Intermolecular interactions between gZiPro and selected compounds were detected by using BLI technology on the ForteBio Octet Red system (Pall FortéBio, Inc, Menlo Park, USA). The biotinylated protein gZipro was immobilized onto super streptavidin biosensors (Pall FortéBio Inc.), and then blocked with biocytin (Sigma). The captured biosensors were individually dipped into wells containing various concentrations of ZP10 (3.125, 6.25, 12.5, 25, and 50 µM). Sensorgrams were double-referenced, corrected for solvent effects, and then fit using OctetRed user software (Pall FortéBio Inc.). The binding profile of each sample was summarized as an “nm shift” (the wavelength or spectral shift in nanometers), which represented the difference between the start and end of the kinetic cycle. Steady-state and kinetic responses were fit to a simple binding model to obtain values for association (K_on_) and dissociation (K_off_) rate constants and the equilibrium dissociation constant (K_d_). The Langmuir’s 1:1 binding model was applied to fit the interferometry data.

### Cells and Virus

African green monkey kidney cells (Vero E6) were maintained in Dulbecco’s modified Eagle’s medium (DMEM) with 10% (v/v) fetal bovine serum (FBS; Invitrogen). Human glioblastoma cell lines U-87 MG were maintained in Eagle’s Minimum Essential Medium (EMEM) with 10% (v/v) fetal bovine serum (FBS; Invitrogen). HEK293T cells were maintained in Dulbecco’s modified Eagle’s medium (DMEM) with 10% (v/v) fetal bovine serum (FBS; Invitrogen). PLCal_ZV Zika virus strain (GeneBank accession number: KF993678.1) was provided kindly by Dr. Chen Liang (McGill University). Virus titers were determined on Vero E6 cells and represented as the median tissue culture infective dose (TCID_50_).

### Cytotoxicity Assay

Cell viability was performed by Cell Counting Kit-8 (CCK-8) (Beyotime, China). Typically, 8×10^4^ cells/ml Vero E6 cells were seeded in the wells of 96-well plate at 100 μl/well and grown for 24 h. The compound 1 μl was added to the cells, after which cells were infected with ZIKV at MOI of 0.05. Ribavirin and DMSO were used as positive and negative control, respectively. After a further incubation for 96 h at 37°C, cell supernatants were replaced with 110 μl fresh medium containing 10 μl CCK-8 reagent and incubated for 1.5 h at 37°C with 5% CO_2_. The absorbance at 450 nm was subsequently measured using EnSpire 2300 Multilable Reader (PerkinElmer). The inhibition rate of the tested compounds was calculated with the following equation:

$$\text{Inhibition}\;\text{rate}\%=100-\left[\frac{OD_{tested\;compound}-OD_{mock-infected\;cells}}{OD_{infected\;cells}-OD_{mock-infected\;cells}}\right]\times 100\%$$

### qRT-PCR

To determine the viral RNA number by qRT-PCR, total intracellular RNAs were extracted from the infected cells (control and treated) by treatment with TRIzol reagent (Invitrogen, USA) according to the manufacturer’s protocol. The cDNA was synthesized using Primescript RT Master Kit (Takara, Japan). Level of viral RNA was determined by performing qRT-PCR analysis using SYBR premix Ex Taq II kit (Takara) according to the instructions. The primer sequences used for the Zika virus genes are 5’-CCACGCACTGATAACAT-3’ (forward) and 5’-AAGTAGCAAGGCCTGCTCT-3’ (reverse). For quantification, the 2^−ΔΔCt^ method was used to calculate the relative RNA levels against GAPDH.

### Western Blot

Vero E6 cells were seeded at a density of 2×10^5^ cells per well in 6-well plate. After overnight incubation, 2 µl DMSO (Sigma-Aldrich) or 2 µl chemical compounds were added to each well at the designated concentration. Then cells were infected with ZIKV at MOI of 0.05. Ribavirin and DMSO were used as positive and negative control, respectively. After a further incubation for 72 h at 37°C, cells were lysed with 100 μl of lysis buffer (50mM Tris, 150mM NaCl, 1% NP-40, 0.5% sodium deoxycholate, pH 7.4). Cell lysate was subjected to denaturing gel electrophoresis with 10% Bis-Tris Gel. The proteins were transferred onto PVDF membranes (Millipore, USA) and immunoblotted with indicated antibodies. The following antibodies were used for the detection of proteins: Anti-NS2B antibody (GTX133308, 1:1,000, GeneTex), Anti-NS3 antibody(GTX133320, 1:1,000, GeneTex), Anti-NS4A antibody (GTX133704, 1:1,000, GeneTex), Anti-prM antibody, Anti-β actin antibody (mAbcam 8224, 1:5000, Abcam) was used as a loading control. Proteins were visualized using horseradish peroxidase-conjugated secondary antibodies (Zhongshan Jinqiao Biotechnology, China). Chemiluminescent signals were produced by the Chemiluminescent HRP Substrate (Millipore, USA). Blots were imaged using the Gel Doc XR+ molecular imager (Bio-Rad, Hercules, USA).

Quantification of signals on Western blots was done using the Image pro 10 (Media cybernetics, USA) Imaging and Processing Analysis Software with signaling intensity normalized to loading control. IRDye800CW Goat anti-Mouse 926-32210 and IRDye680RD Goat anti-Rabbit secondary antibodies were used at 1:20,000 dilution (LI-COR, Lincoln, NE). Membranes were imaged and quantified in 800 nm channelsin the Odyssey Infrared Imaging System using Odyssey V3.0 software (LI-COR, Lincoln, NE).

### Time-of-Drug-Addition Assay

For the pre-treatment group, Vero E6 cells were seeded 12-well plates (2.5×10^5^ cells/well). Compound ZP10 (25 μM) or ZIKV neutralization antibody (0.1 μg/ml, a kind gift from Dr. Fu Gao, Chinese Academy of Sciences) were first incubated with ZIKV (MOI=0.05) for 1 h at 37 °C under 5% CO_2_. Next, Vero E6 cells were washed with PBS once before the addition of mixture. The 600 μl mixture was added into cells for 1 h at 4°C. The cells were washed with PBS three times before added DMEM medium containing ZP10 25 μM. Infected cells were collected at 24 hpi. The cell lysate was collected for viral RNA copies measurement using qRT-PCR. DMSO was included as negative control. The experiments were performed in triplicate and repeated twice for confirmation. For the post-treatment group, compound ZP10 (25 μM) or neutralization antibody (0.1 μg/ml) only added in to cells after the viral adsorption stage (0 hpi), followed by incubation of the cells incubated until 24 hpi.

### Proteinase Cleavage Assays

Minimal self-cleaving proteinase NS2B/3pro was cloned in pcDNA3.1(+) through Gibson assembly Master Mix (NEB, USA). The proteinase active-site mutation, NS3-S135A, was produced by Mut Express II Fast Mutagenesis Kit V2 (Vazyme, China). All constructs were confirmed by sequencing. Transfection was performed with lipofectamine 2000 (Invitrogen, USA) according to the manufacturer’s instructions. After 48h transfection, cell lysates were subjected to western blot.

### Immunofluorescence

Vero E6 cells were infected with ZIKV at MOI=0.05 and incubated at 37°Cfor 72 h. Cells were washed thrice with ice-cold PBS, fixed in 4% paraformaldehyde for 10 min, and permeabilized in 0.2% Triton X-100 for 10 min. The cells were blocked with 1% bovine serum albumin (BSA) for 1 h. Cells were incubated with ZIKV anti-Envelop antibody (1:1,000) (Genetex, USA) at room temperature for 2 h and washed three times with PBS (5 min for each wash). The cells incubated with Alexa-conjugated secondary antibodies (Invitrogen) (1:1,000) for 1 h at room temperature, after which the cells were washed three times with PBS. The cells were then stained with DAPI and mounted using Mounting Medium. Fluorescence images were visualized using the Zeiss LSM 710 confocal microscope (Oberkochen, Germany). Calculation of green immunofluorescence intensity was done using the Image pro 10 (Media cybernetics, USA) Imaging and Processing Analysis Software with signaling intensity normalized to loading control.

### Molecular Docking

For docking on the ZIKVpro, the crystal structure of gZiPpro was used (PDB code: 5LC0). The crystal structures of ZIKVpro in complex with a peptidomimetic boronic-acid inhibitor (PDB code: 5LC0) was prepared using the Protein Preparation Wizard. Since 5LC0 is a crystallographic dimer, a single NS2B-NS3 construct (chain A) was selected for docking. For the docking, ZP10 was docked into the binding site of receptor using MOE software. The active site for ZIKV was defined as the shell of residues within 10 Å around the catalytic residue Ser135 in the NS3 protease domain. The default parameters were used. The protease was held rigid during the docking process, while ZP10 was allowed to be flexible. Docking simulations was performed using a grid box with dimensions of 45×45×45 Å, a search space of 30 binding modes. The best docking pose for ZP10 was selected on the basis of the lowest energy docked conformation.

### Statistical Analysis

All experiments were performed in triplicates unless specified otherwise. Western blots were quantified using the Bio-Rad Gel Doc EZ system and Image Lab software #1709690 (Bio-Rad).

The statistical analyses were conducted using Graphpad Prism 7.00 software. The significance level among two groups was calculated using two-tailed unpaired t-test. The significance level among multiple groups was identified by one-way ANOVA with Tukey *post hoc* test. p-value < 0.05 was considered to show a significant difference.

Z’ factor (Z’) was calculated using the following equation: Z’ =1-3(δ_p_ + δ_n_)/|μ_p_ -μ_n_|, where μ_p_ and μ_n_ represent mean values of wells treated by 0.1% DMSO and wells treated by 100 μM myricetin; δ_p_ and δ_n_ are the standard deviations. Coefficient of variation (CV) was calculated using the following equation: CV (%) = δ_p_/μ_p_ × 100. Ratio of signal to noise, a factor to represent signal strength was calculated as the following: $S/N=(\mu_{p}-\mu_{n})/{\left(\delta_{p}^{2}+\delta_{n}^{2}\right)}^{1/2}$.

## Data Availability Statement

The datasets generated for this study are available on request to the corresponding authors.

## Author Contributions

XC and RuZ performed the experiments and analyzed the data. CH and YZ helped to set up the assay methodology. JZ, XL, JW, JD, YZ, and SC provided the resource and partial funding supports. JZ designed the experiments and wrote the original manuscript. JZ and SC reviewed and edited the manuscript. All authors contributed to the article and approved the submitted version.

## Funding

This work was in part supported by the National key research and development program (2018YFE0107600 ZJM), the Nature Science Foundation of China (81672559), the CAMS Innovation Fund for Medical Sciences (CAMS 2018-I2M-3-004 CS), and the National Mega-Project for Significant new drug discovery (2018ZX09711003-002-002, ZJM).

## Conflict of Interest

The authors declare that the research was conducted in the absence of any commercial or financial relationships that could be construed as a potential conflict of interest.
