# Supplementary material for: Identification of Theaflavin-3,3’-Digallate as a Novel Zika Virus Protease Inhibitor
Source: Front Pharmacol. 2020 Oct 21;11:514313. doi: 10.3389/fphar.2020.514313 (PMC7609463; doi:10.3389/fphar.2020.514313)
Supplement: Supplementary file 1 [file Presentation_1.pdf]

Supplementary materials

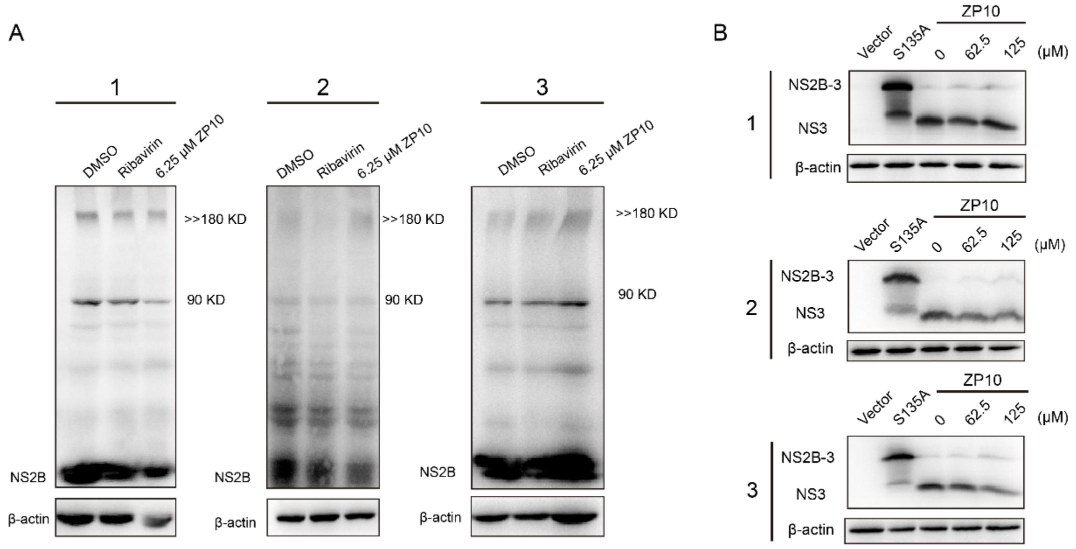

PP/preNS2B-3

	1	2	3	SD
DMSO	0.659	0.880	0.599	0.121
Ribavirin	0.449	0.869	0.385	0.214
ZP10	1.271	1.623	1.133	0.206

Normalized NS2B-3/NS3

	1	2	3	SD
62.5 μM ZP10	1.595	1.499	1.604	0.048
125 μM ZP10	2.003	1.960	2.018	0.024

Figure 1. A) The triplicates of figure 6A used for quantification. **B)** The replicates of Figure 6D used for quantification.
